# Spitting Performance Parameters and Their Biomechanical Implications in the Spitting Spider, *Scytodes thoracica*

**DOI:** 10.1673/031.009.6201

**Published:** 2009-11-19

**Authors:** Robert B. Suter, Gail E. Stratton

**Affiliations:** ^1^ Department of Biology, Vassar College, Poughkeepsie, NY 12604, USA; ^2^ Department of Biology, University of Mississippi, University, MS 38677, USA

**Keywords:** spider predation, fluid flow, hydrodynamics, oscillation, spider morphology

## Abstract

Spitting spiders *Scytodes* spp. subdue prey by entangling them at a distance with a mixture of silk, glue, and venom. Using high-speed videography and differential interference contrast microscopy, the performance parameters involved in spit ejection by *Scytodes thoracica* (Araneae, Scytodidae) were measured. These will ultimately need to be explained in biomechanical and fluid dynamic terms. It was found that the ejection of “spit” from the opening of the venom duct (near the proximal end of the fang) was orderly. It resulted in a pattern that scanned along a lateral-medial axis (due to fang oscillations) while traversing from ventral to dorsal (due to cheliceral elevation). Each lateral-to-medial sweep of a fang produced silk-borne beads of glue that were not present during each subsequent medial-to-lateral sweep. The ejection of “spit” was very rapid. A full scan (5–57 fang cycles, one upsweep of a chelicera) typically occupied less than 30 ms and involved fang oscillations at 278–1781 Hz. Ejection velocities were measured as high as 28.8 m/s. The “spit” was contractile. During the 0.2 s following ejection, silk shortened by 40–60% and the product of a full scan by both of the chelicerae could exert an aggregate contractile force of 0.1 – 0.3 mN. Based on these parameters, hypotheses are described concerning the biomechanical and fluid dynamic processes that could enable this kind of material ejection.

## Introduction

Spitting spiders (*Scytodes* spp.) are so named because of their remarkable habit of ejecting from their fangs a mixture of silk, venom, and glue onto potential prey (Monterosso 1927; MacAlister 1960). They also produce silk through posterior spinnerets, as do other spiders, and at least some species use a web in capturing prey (Nentwig 1985; Gilbert and Rayor 1985), but these more orthodox capabilities are not the subjects of this paper.

Subduing prey or deterring predators by spitting upon them apparently occurs exclusively in spiders from the families Scytodidae and Oxyopidae. In the latter group, green lynx spiders, *Peucetia viridans* sometimes spray a liquid from their fangs in self-defense or when protecting eggs (Fink 1984); this behavior has not been reported in other oxyopids. In groups other than arachnids, defensive or aggressive spitting, or spitting as a part of prey capture, occurs in insects (e.g., prey-capture nets constructed from oral secretions by hydropsychid, philopotamid, and polycentropid caddisfly larvae: Brown et al. 2004; Edler and Georgian 2004), reptiles (e.g., spitting cobras: Young et al. 2004) and mammals (Camelidae: Hoffman 1993). What makes the spitting spiders particularly interesting from a biomechanical perspective is that their spit, despite being a mix of viscid fluids and silk, is ejected very rapidly and in a highly organized pattern through a very small orifice. This orifice is located near the proximal end of the disproportionately small fang that is borne by a disproportionately small chelicera (Suter and Stratton 2005). These allometries may be related to the biomechanics of spitting.

Here the performance parameters of spitting by *Scytodes thoracica* (Araneae, Scytodidae) are described and quantified including the pattern and speed of spit ejection, and the mechanical properties of the spit that allow formulation of hypotheses about the underlying biomechanics. This required (1) eliciting spitting repeatedly and under controlled circumstances; (2) measuring both the linear and the oscillatory dynamics of spitting; and (3) elucidating some aspects of the anatomy of the spitting apparatus.

## Materials and Methods

### Spiders

**Figure 1.**
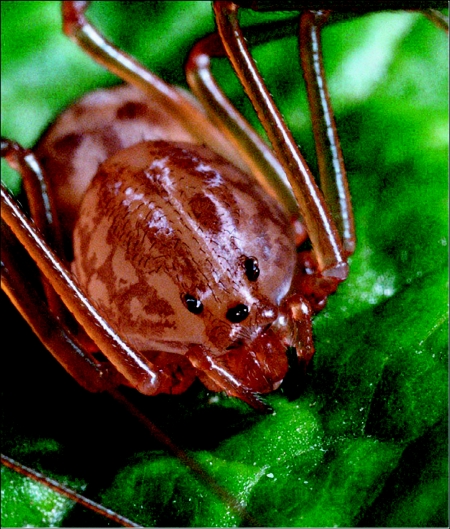
Frontal view of *Scytodes thoracica* showing two characteristics that typify the spitting spiders: the domed cephalothorax containing disproportionately large venom glands and the diminutive chelicerae.

The *S. thoracica* spiders used in this study (Figure 1) were sent to us by arachnologists working at a variety of locations in the southern half of the United States (see acknowledgements). They were maintained individually in 260-ml polystyrene foam cups, covered by lids to 100 X 15-mm plastic Petri dishes, on a diet of vinegar flies (*Drosophila melanogaster*) supplemented occasionally with wild-caught small insects. Both humidification and drinking water were provided by way of a wick that passed through the bottom of each cup and into a reservoir of distilled water. Temperature in the laboratory varied between 19 and 22 °C and the photoperiod experienced by the spiders was erratic but each day included a period of darkness from 2000 h in the evening to 0500 h in the morning.

### Immobilization

**Figure 2.**
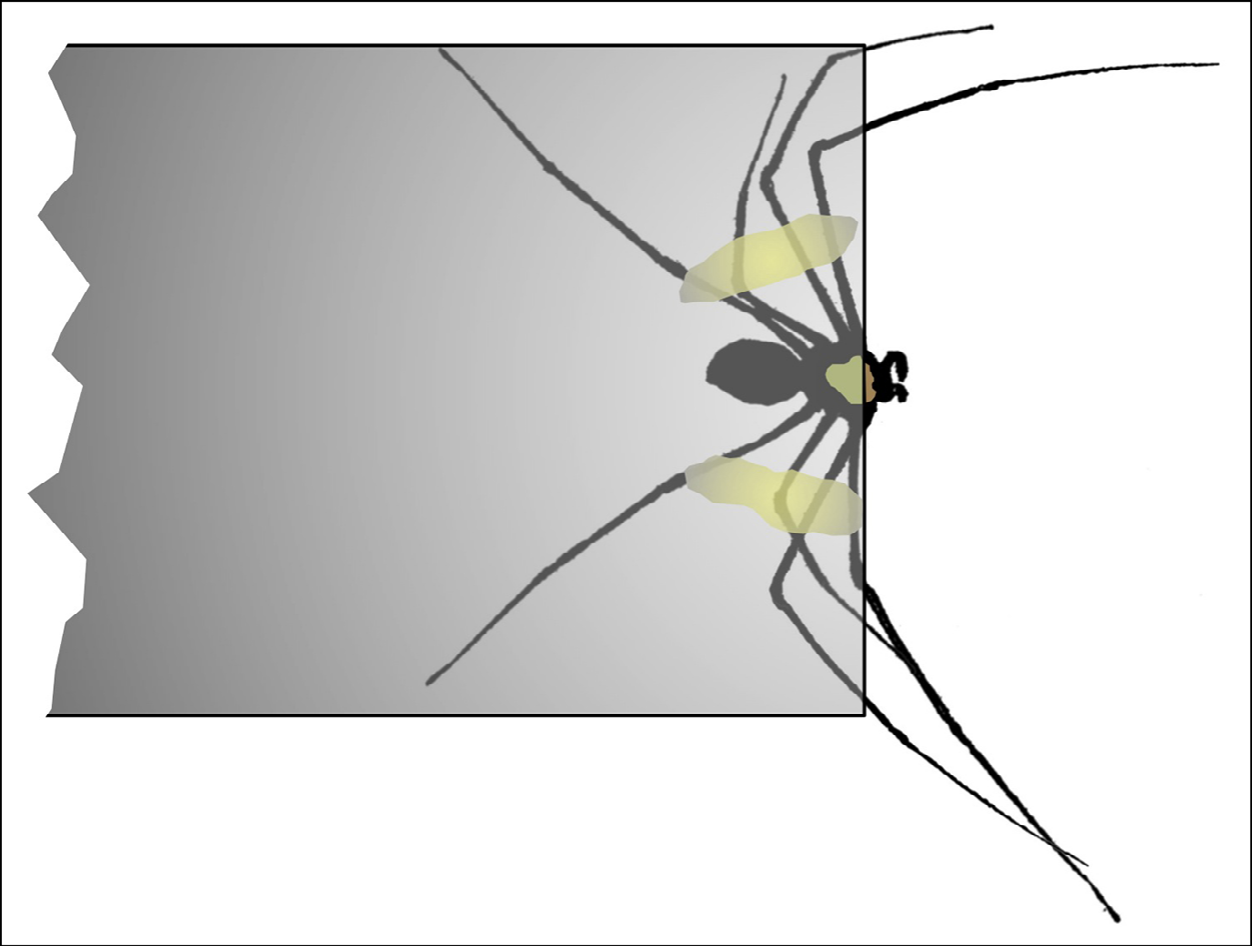
Diagrammatic view of *Scytodes thoracica* affixed to the end of a microscope slide in preparation for eliciting expectoration under controlled conditions. The view is looking through the slide at the dorsal surface of the spider. Three tan patches, one on the cephalothorax and the others on the legs, represent the epoxy used to immobilize the spider.

The exploration of spitting behavior of the spiders required immobilized subjects that could be focused on reliably with a high-speed video camera and from which material extruded during spitting could be captured. Each individual was anaesthetized in a vial filled with CO2 and then placed on its back at the end of a glass microscope slide (25 × 75 mm). Quick-setting epoxy glue was used to anchor the dorsal surface of the cephalothorax and all eight legs to the slide. The spider was thus effectively immobilized, with its ventral surface exposed and with a small fraction of the cephalothorax protruding beyond the edge of the slide (Figure 2). This form of immobilization could alter the parameters being measured.

### Eliciting expectoration

Spitting by unrestrained spiders occurs after stimulation, perhaps not only tactile but also visual and chemosensory, and may be either defensive or predatory (Gilbert and Rayor 1985; Jackson and Pollard 2001). The usual complex of stimuli and context could be circumvented by gently stroking the ventral surface of the cephalothorax with the barb from the feather of a passerine bird. The barbs we used were approximately 1 cm long, mounted at the end of a dissecting needle to facilitate manipulation. Using this method, spitting could be reliably elicited when the spider was either with its ventral surface down (relative to the Earth) or up, when its cephalothorax was in the dim light (500 lux) or bathed in intense light (15000 kilolux), and when it was facing nothing but air. Spitting was also elicited when it was confronted, at 1.5 cm, by probe wires or the surface of a microscope slide.

### High-speed videography

The core of the video system used was a high-speed motion analysis system (MotionScope S series, Redlake Imaging Corporation, www.redlake.com) that was capable of monochromatic image capture rates up to 1000 frames per second (fps) and effective shutter speeds as fast as 1/5000 s. The camera itself has a “C” mount that allowed the use of a normal video lens or, as was necessary in capturing fang movements, a bellows-mounted, reversed, enlarger lens stopped down to an aperture of f16. Very high intensity illumination was needed under the last of these conditions. Video sequences captured digitally by the motion analysis system were recorded in VHS format at 30 frames/s (JVC HR-S5400U). When played back at 30 fps, movements of the chelicerae, fangs, and silk, slowed to 0.03 or 0.06 normal speed, could be qualitatively analyzed on a video monitor. More detailed measurements (e.g., of cheliceral angular deflection) were made using frame-by-frame analysis in *NIH Image*; this shareware is available from the National Institutes of Health (http://rsb.info.nih.gov/nih-image/).

The spiders or the extruded silk mixture were illuminated using as the primary light source a 30OW projection lamp (ELH, Sylvania) located 40 cm from the subject. All of the light falling on the spider first passed through a 23-mm thick filter (distilled water in a 25 cm^2^ tissue culture flask: Corning, www.corning.com/lifesciences) to remove most of the IR radiation without substantially reducing the visible light levels. To achieve the high intensities needed to image the fangs and chelicerae at high speed, high magnification, and small aperture, a convex lens was placed between the filter and the subject was positioned close to the focal point of the lens.

### Microscopy

A scanning electron microscope (Amray 1200C) was used to look at surface details of the fangs and chelicerae of the spitting spiders, and differential interference contrast microscopy (DICM: Olympus Provis AX70 with UIS optical system and transmitted Koehler illumination, www.olympus.com) to visualize both the material deposited on a slide during expectoration and some features of the fangs and chelicerae when cleared in clove oil. Specimens to be viewed with the scanning electron microscope were killed in 70% EtOH, preserved in 100% EtOH, and then freeze-dried and sputter-coated with gold and palladium in a ratio of 80:20. In preparation for DICM imaging of chelicerae and fangs, the chelicerae were separated from the prosoma of a preserved spider and placed on a microscope slide, bathed in clove oil, under a cover slip.

In preparation for DICM imaging of the products of expectoration, a microscope slide was placed in a plane perpendicular to the long axis of a spider–'s immobilized body, about 1.5 cm in front of the spider, and the spider was induced to spit. Within an hour following the collection of spit, the slide was placed directly on the microscope stage without further treatment and in the absence of a cover slip. The pattern of deposition of fluid globules along spit fibers was analyzed using the Clark-Evans index (Clark and Evans 1954) in which values >1 indicate regular, non-random spacing. Using *NIH Image*, the center-to-center distances were measured between adjacent globules on six strands of silk from a typical spitting episode. These distances are expected to come from a random distribution unless some process giving them order (e.g., clumping or hyper-dispersion) governs their distribution. The Clark-Evans index, in effect, measures deviation from the expected random distribution.

To facilitate sectional views of the internal anatomy of the spider's chelicerae, 1.0 µm-thick serial sections of whole chelicerae were made with a diamond knife after fixation and staining in toluidine blue. Fixation was accomplished in buffered solution containing 2.5% gluteraldehyde and 2.0% paraformaldehyde, followed by post fixation in 2.0% osmium tetroxide, stepwise dehydration culminating in absolute acetone, and embedding in Spurr's low viscosity medium.

**Figure 3.**
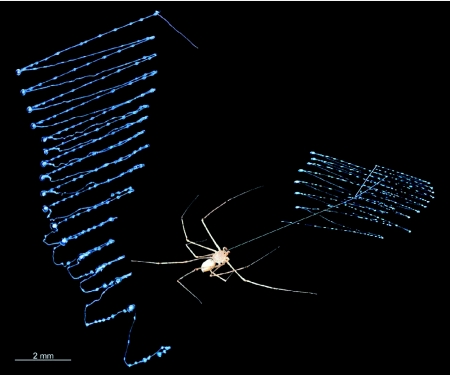
Composite showing (right) *Scytodes thoracica* and its highly organized spit produced as two zig-zag patterns, one from each fang; and showing (left) the enlarged spit pattern produced during one expectoration episode by one left chelicera/fang. The spit patterns were collected on microscope slides and visualized via DICM imaging. The straight line connecting the spider to the spit pattern was added in Photoshop, but it represents a strand of silk that often appears in just that way just after a spitting episode.

### Calculation of extrusion length, volume, velocity, and oscillation frequency

Because the pattern of material deposited during a spitting event is organized and readily visualized (Figure 3; Foelix 1996, Gilbert and Rayor 1985), the length of the material extruded onto microscope slides could be directly measured. For N = 9 individuals and 18 episodes of spitting, the average rate of extrusion (m/s) during a spitting event was calculated by dividing the length of the extruded material by the duration of the event. To determine volume, the length of the deposited material was multiplied by an estimate of the cross sectional area of the silk strands visible in DICM images. The estimation of the cross sectional area involved assumptions that, being untested, warrant skepticism about the resulting values for volumetric rates of extrusion: it was assumed (1) that the silk component of the spit was cylindrical, (2) that the silk itself was the major component, with respect to volume, of the extruded material, and (3) that each of the two visibly different sizes of the silk component was present in about half of each spitting event. The duration of an event was measured in high-speed video sequences, shot from a lateral perspective, in which both the changing angle of the chelicerae and the emerging material could be seen. The regularity of the pattern of deposition, usually described as a zigzag pattern, allowed the calculation of the oscillation frequency (Hz) by dividing the number of deposited zigzags by the duration of the event.

### Force measurements

**Figure 4.**
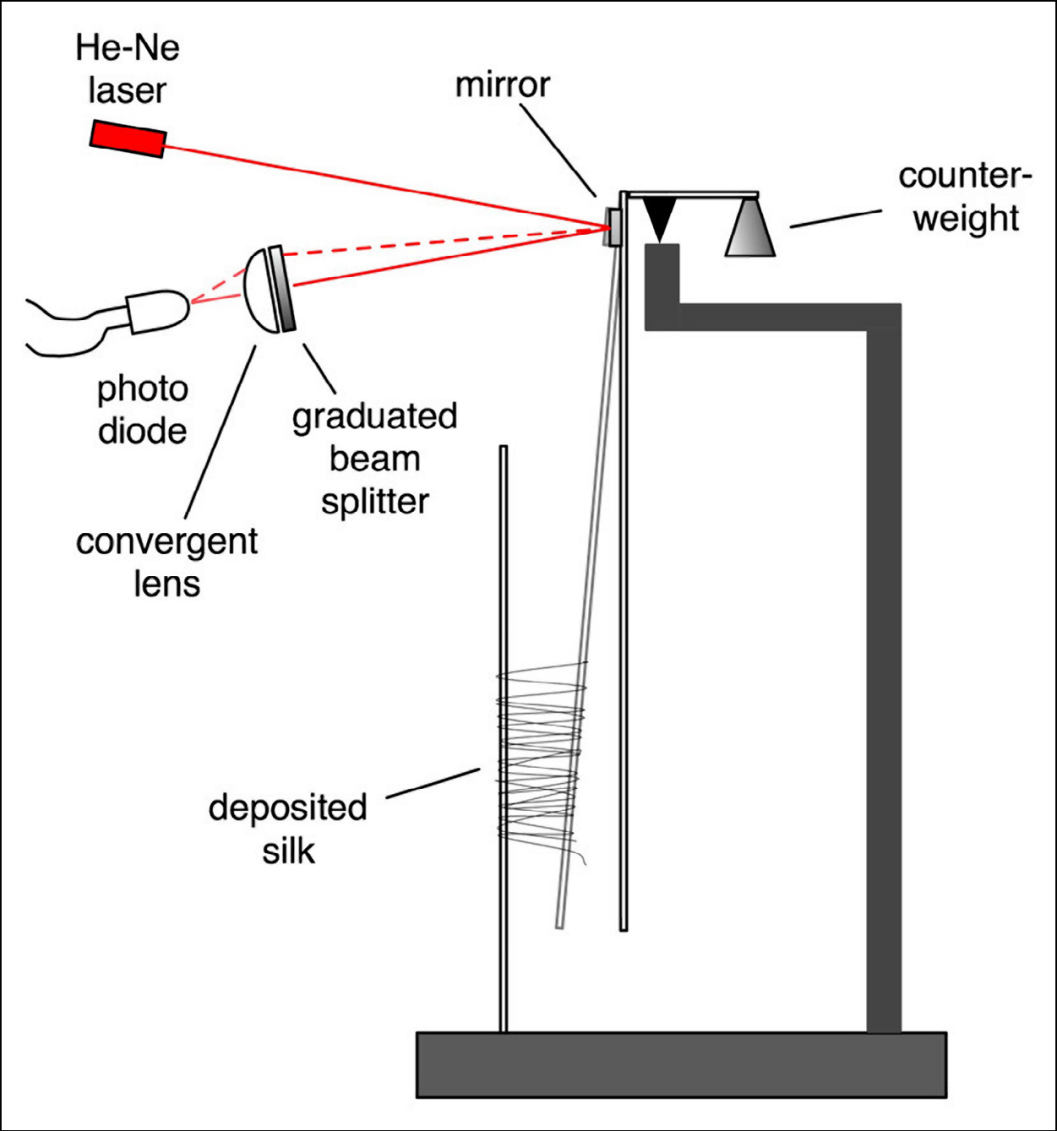
Diagrammatic representation of the instrument used to measure the force generated by contraction of the silk ejected during one spitting episode. In this view, the spider is located behind the plane of the image, facing the viewer through the deposited silk (see Video 4). Before expectoration, the two collection wires are vertical; silk contraction causes the movable wire to be pulled toward the fixed wire, in turn deflecting the laser beam to a higher position on the graduated beam splitter and allowing more of the laser light to stimulate the photo diode sensor. Changes in output (mV) from the photo diode are recorded in digital form for later analysis.

Once it was clear from the present study that a silk-like component was part of the spit of *S. thoracica*, it was necessary to determine whether this component was contractile. A force probe was designed (Figure 4) the active part of which was two vertical wires 5 mm apart, one fixed and the other effectively a pendulum. When a spider (N=5) spat upon the two wires, the amount of deflection of the movable wire toward the fixed wire was a measure of the force exerted by the contraction of the silk-like component. The deflection of the wire caused an identical angular deflection of a small front-surface mirror mounted on the movable wire at the fulcrum of the pendulum, the prism's motion deflected a laser beam, and the movement of the laser beam was transduced to an electrical signal as it passed through a graduated beam splitter and onto a photodiode. The resulting electrical signal was digitized using Vernier LabPro (www.vernier.com) hardware and software.

## Results

Spitting spiders, *S. thoracica*, ejected material from their venom ducts at velocities as high as 28 m/s (mean ± SE: 10.32 ± 1.99 m/s) while the fangs oscillated at frequencies up to 1700 Hz (826.3 ± 102.7 Hz), depositing material in an orderly zigzag pattern (Figure 3), all in less than 35 ms (25.2 ± 1.4 ms; Table 1). During a spitting episode, the lateral-medial oscillation (Video 1) of the fangs was accompanied by ventral-to-dorsal extension of the chelicerae (Videos 1, 2) and the combination of these two actions accounts for the zigzag pattern of spit deposition. While the spit was airborne, the zigzag pattern was also evident (Video 3). To direct the spit in a zigzag at the mean 826 Hz shown in Table 1, the fang must achieve the same oscillation frequency. Because one full translation of the fang covers about 75° (Video 1), the fang must have had an average angular velocity of about 123,900°/s or about 2,160 radians/s.

**Video 1.**
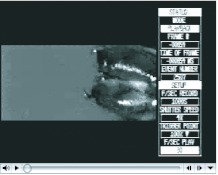
Two episodes of “spit” ejection by *Scytodes thoracica*, captured at 1000 frames per second. See text for details. URL:http://www.insectscience.org/9.62/ref/i1536-2442-9-58-v01.avi

**Video 2.**
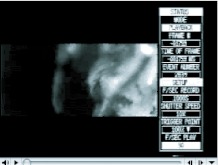
Five episodes of expectoration by *Scytodes thoracica*, captured at 1000 frames per second. The lateral view emphasizes the ventral-to-dorsal sweep of the chelicerae during expectoration; video of sweeps like these were used to determine the durations of individual “spitting” events. URL:http://www.insectscience.org/9.62/ref/i1536-2442-9-58-v02.avi

**Video 3.**
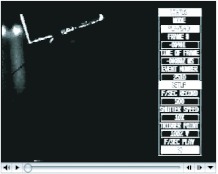
Two episodes of “spit” ejection by *Scytodes thoracica*, viewed from above, in the absence of a solid target for the ejected material. See Figure 2 for a representation of the position of the spider during events such as these. URL:http://www.insectscience.org/9.62/ref/i1536-2442-9-58-v03.avi

**Table 1.**
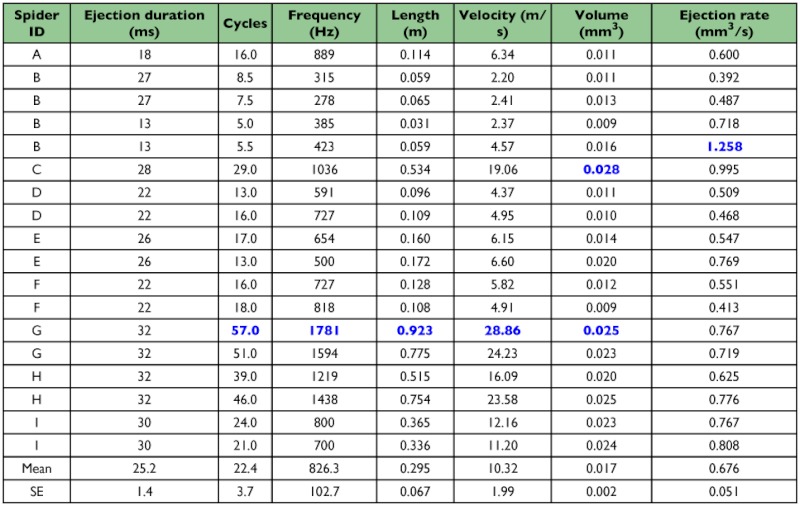
Analyses of ejection rates based upon ejection duration (measurements from HS video), length of product (measurements from materials deposited on microscope slides), and thread diameters (estimates from DICM images). Maximum values are identified by blue font. Multiple entries from the same spider indicate pairs of ejections (left and right) that occurred during the same event.

**Video 4.**
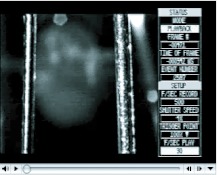
The effect of silk deposition on a transducer designed to measure the force of contraction of the silk. The spider, out of focus, is in the background facing the camera. See text for details and Figure 4 for a description of the transducer. URL:http://www.insectscience.org/9.62/ref/i1536-2442-9-58-v04.avi

**Figure 5.**
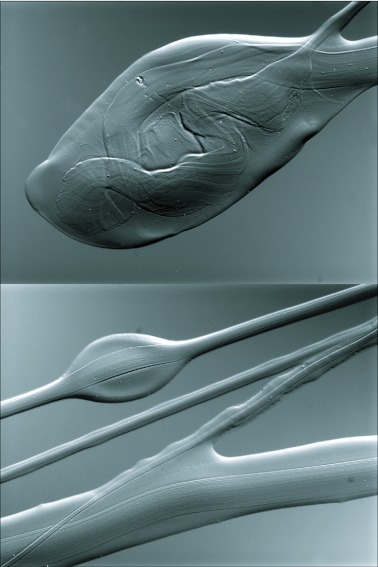
DICM images of spit of *Scytodes thoracica* deposited on a microscope slide and photographed within an hour of expectoration. In both images, a fiber-like material can be seen immersed in what appears to be a viscous liquid. Top — a terminal bleb of fluid and fiber typically found at the distal transitions between a zig and a zag (see Figure 3). Bottom —part of a spit pattern where materials from both right and left fangs overlap. Differences between fiber diameters and amounts of fluid are conspicuous, as is the fluid droplet that is characteristic of strands of spit deposited during inward (mediad) excursions of a fang (see Figure 3).

Inspection of the spit deposited on a microscope slide revealed that it was composed of liquid and fiber. The liquid remained entrained to the fiber during ejection and the two materials were deposited together (Figures 3, 5; Videos 3, 4). Ejection of the fluid part of the spit was not uniform (see also Foelix 1996): the medial-to-lateral phase of each zigzag consisted of a fiber surrounded by relatively little fluid and the fluid itself remained evenly distributed along the fiber; in contrast, the lateral-to-medial phase consisted of a fiber bearing visible globules of fluid arrayed in a significantly non-random, regular pattern (N=6 lateral-to-medial sweeps; average Clark-Evans index for the 6 sweeps = 1.91; P<0.01 for each sweep).

**Figure 6.**
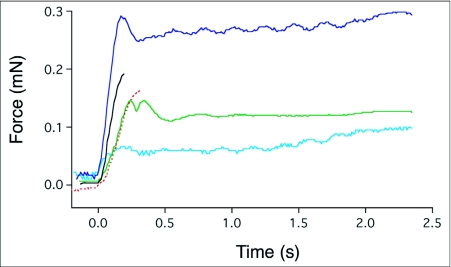
The force of contraction, measured with the transducer represented in Figure 4, of the silk deposited during five independent spitting episodes by *Scytodes thoracica*. Nearly all of the contraction occurs in the first 0.2 s after deposition and, depending in part on the number of silk strands involved, can reach an aggregate force of nearly 0.3 mN. The dynamics of this process can be viewed in Video 4.

When the spider's target was a pair of wires instead of a flat surface, the fibrous spit wrapped around the target and, in the subsequent 0.3 s or so, contracted (Video 4). The contraction exerted a peak aggregate force between 0.05 and 0.3 mN (Figure 6) that was dependent, in part, on the number of strands of fiber connecting the two wires of the force transducer.

**Figure 7.**
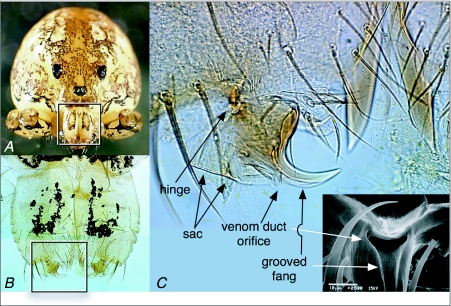
External anatomy of the chelicerae and fangs of *Scytodes thoracica*. (A) Frontal view of *S. thoracica*. The legs and pedipalps of this specimen were removed so that they didn't obscure the face. The box outlines the region shown in B. (B) Anterior view of the chelicerae and fangs, both cleared with clove oil. The box outlines the region shown in C. (C) DICM (differential interference contrast microscopy) image of the fang and associated structures of a spitting spider after clearing with clove oil. Immediately above the hinge are two arrays of white, curved lines; these are the lyriform sense organs presumed to provide the spider with proprioceptive information about fang position. The DICM view is of the anterior surface of the distal portion of the spider's right chelicera, and thus shows the fang itself in side view. The inset, a scanning electron microscope image, gives an end-on view of the opening to the venom duct, with the sac above it and a groove leading from the orifice to near the tip of the fang.

**Figure 8.**
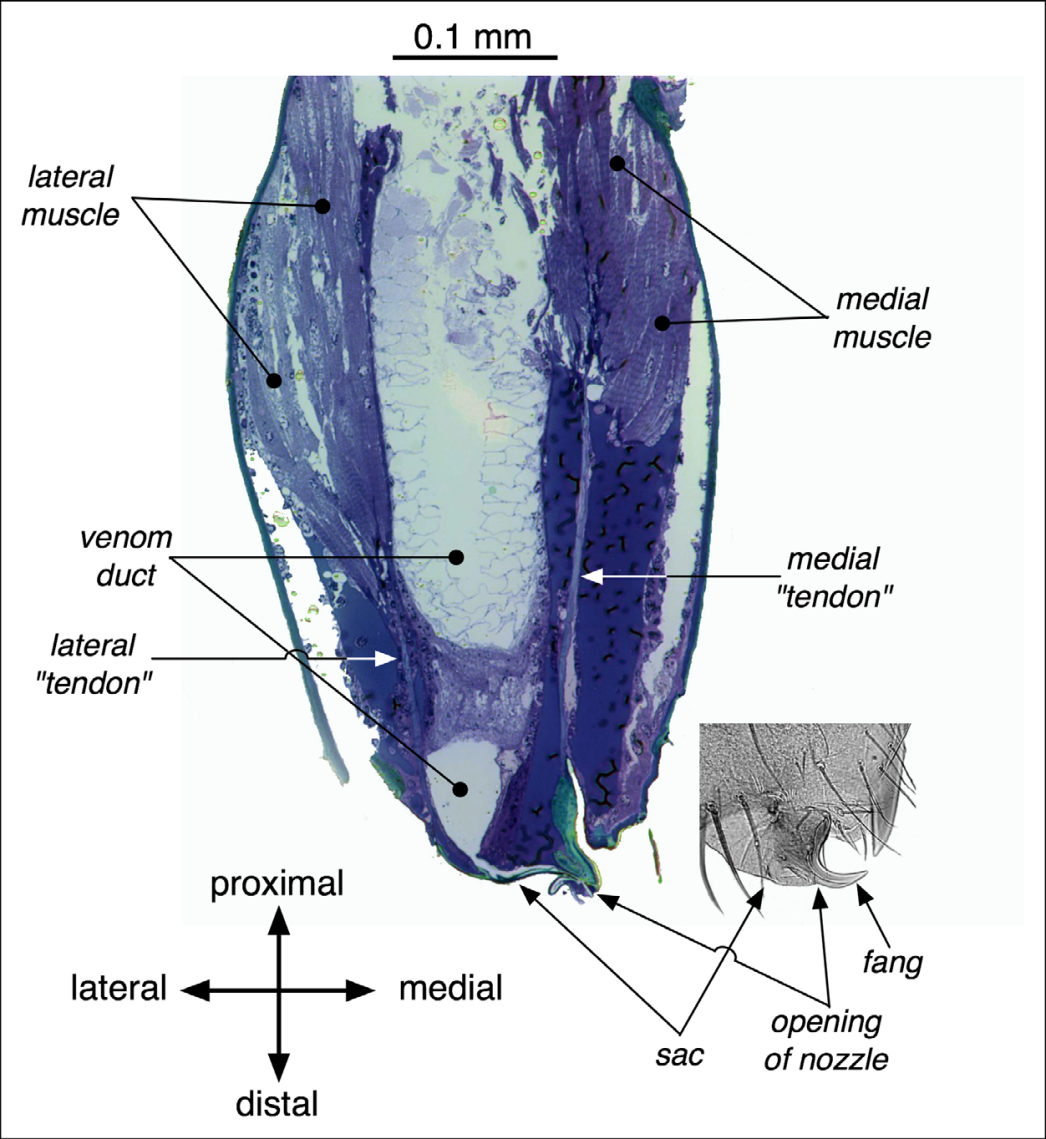
Internal features visible in a stained section of *Scytodes thoracica*'s right chelicera (same orientation as in Figure 7). The following are worth noting: (1) the muscles are those that move the fang and that may be responsible for some of the elasticity involved in the high-frequency oscillation of the fang during spitting (see discussion); (2) the venom duct is broad throughout its length in the chelicera, only narrowing, abruptly, just proximal to the opening on the fang; and (3) the sac, somewhat bellows-like, may facilitate rapid fang oscillation, because of its flexibility, while allowing the venom duct to remain expanded as far as the base of the fang.

The anatomical features that support the spitting performance described above include properties of the fangs and their surrounding tissues (Figure 7). Of particular interest, in this regard, are the hinge and its accompanying lyriform proprioceptive organs, the orifice and the sac that is just lateral to it, and the diminutive (Suter and Stratton, 2005) grooved fang itself. The hinge both constrains the degrees of freedom of the fang and allows the oscillation of the fang and its associated structures at the high angular velocities given above. The orifice shown in Figure 7, that of an adult female *S. thoracica*, had a width of 0.014 mm, twice as wide as the most slender spit fibers measured (0.007 mm) but much narrower than the largest fibers (0.066 mm). Stained sections of the chelicerae of *S. thoracica* revealed that the venom duct, which conveys the spit's constituents to the fang, occupied much of the volume of the chelicera (Figure 8). Figure 8 also shows the location and orientation of two cheliceral muscles that have tendon-like connections, one to the tissue comprising the outside of the sac and the other to the medial aspect of the fang.

## Discussion

These data reveal a strongly organized and at the same time impressively rapid process of expectoration in spitting spiders, a process made possible by a suite of morphological characteristics that are probably unique to the Scytodidae. In what follows, we explore what is known about the material that is ejected during spitting, comment on the morphology of the ejection apparatus, and offer two hypotheses, either of which could adequately explain the forces underlying the rapidity and efficacy of the process of expectoration.

### Silk and its ability to contract

The material ejected from a spitting spider's venom duct contains long, continuous, fibrous strands that are visible under DICM imaging and, with proper back-lighting, in the images captured by high-speed videography. These observations alone would not justify labeling the fibers as “silk.” On the other hand, Kovoor and Zylberberg's (1972) elegant ultrastructural studies of the venom glands of *S. delicatula* suggest that the paracrystalline precursors to the fibers, stored in the venom glands, strongly resemble the paracrystals in the abdominal silk glands of spiders. Given the presence of the genes necessary for abdominal silk production in these spitting spiders, the ultrastructural similarities, and the silk-like appearance of the fibrous material in the spit, we conjecture that the fibers are some form of silk. Continuing work is aimed at determining whether the cephalothoracic silk and the silks that emerge from the spinnerets are indeed homologous.

The literature on the effects of spitting spider spit on their insect prey suggests that the spit, at least in some species, contains venom and that this is responsible for the immobilization of prey (Millot 1930; Nentwig 1985). This assertion is contradicted by the recent work of Clements and Li (2005) in which the spit was found to have no toxic effects on prey when applied externally. Our findings suggest an alternative model for the immobilization of prey: the contractile silken component of the spit, either through a drying process or via some other biophysical mechanism, when combined with the adhesive properties of the liquid in the spit, immobilize the prey item through contraction and adhesion. By rough analogy, the biomechanics of this kind of immobilization may resemble what happens to an insect when it is attacked by *Deinopis* spp. (Deinopidae) with its momentarily stretched net of cribellate silk (Coddington and Sobrevila 1987).

### Morphology

To eject material through a small orifice at high velocity requires (a) a pressure differential from inside to outside and (b) a fluid that has sufficiently low viscosity to flow rapidly at the available pressure (Vogel 1994; Denny 1993). Moreover, to sustain an ejection requires that (c) adequate flow through the ducts leading to the orifice must be possible, and the production of a cohesive stream of ejected material as opposed a spray requires (d) the right combination of orifice /nozzle shape and properties of the fluid.

Although the hydrostatic pressure in the prosoma of spiders (a, above) is already high, providing the power to extend the appendages (Foelix 1996; Parry and Brown 1959), it may be that the pressure must be boosted to enable the rapid expulsion of “spit” that we have measured. Millot (1949, in Foelix 1996) asserted that this is the case, and noted that it is an abrupt contraction of the muscles along the posterior margin of the prosoma that compresses the prosoma and thereby raises the hydrostatic pressure. And because the rate of fluid flow in a tube (c, above) rises in proportion to the tube's radius to the 4^th^ power (Vogel 1994; Denny 1993), it comes as no surprise to observe that the venom duct in spitting spiders is substantially larger, relative to the size of the chelicera (Figure 8), than what is typical for other spiders (Foelix 1996; see also Suter and Stratton 2005). The expanded venom duct extends as far as the base of the fang itself, forming at that point a sac (Figure 8) that may also serve to increase the range of flexibility of the fang during oscillation.

The properties of the materials ejected during spitting (b and d, above), and especially of those materials when they are still behind the orifice and, presumably, in liquid form, remain largely unknown. If future studies show that the silk from the venom glands of the spitting spiders is homologous with the silk produced by the opisthosomal silk glands during web spinning (Kerkam et al. 1991), that will give us a place to start. Since the spit is a combination of silk and fluid once it is ejected (Figure 5), it will be a challenge to understand the spit's ability to persist in air as a single strand (fluid entrained on silk) as opposed to a stream of liquid surrounded by spray (see Vogel 1994, concerning the morphology of the nozzle and its influence on stream characteristics). For a more technical treatment of some of the flow issues that apply to the current situation, see Guyon et al. (2001).

### Expectoration as a process

Details of the deposited spit (Figure 3) indicate an asymmetry in delivery, with less material being extruded during fang extension and more material being extruded during flexion. What is currently know about this system does not allow distinguishing between two conceptually different origins of the asymmetry: either it arises as a consequence of the mechanical processes that propel the fangs during spitting or it is an integral part of a hydrodynamic mechanism that drives the fang oscillations. And that conundrum leads directly to the more central question: What forces might cause the high-frequency oscillations of the fangs (Table 1)?

Oscillation frequencies much higher than 500 Hz in biological systems are often caused by the interplay between an energetic phenomenon (e.g., fluid flow or muscle contraction) and an elastic structure with a high resonant frequency (e.g., the mammalian vocal cord or avian syrinx, or a fly's wing/thorax). This kind of interplay is necessary because muscles have limited contraction and relaxation speeds, leading to constrained contraction frequencies (Syme and Josephson 2002, and references therein).

To account for oscillation frequencies as high as those seen repeatedly during spitting by scytodid spiders (Table 1), there appear to be only two categories of possible mechanisms. In one, muscle firing and effector (fang) motion are *asynchronous* but coupled, so that one muscle twitch drives multiple fang oscillations, a mechanism analogous to what drives the high frequency wing beats of small flies (Josephson et al. 2000). In the other, muscles play no direct role at all in effecting the oscillation; instead, fluid flowing past or through a structure that has some elastic properties causes the structure to oscillate. For example, the vibrations of a song bird's syrinx (excluding, in this consideration, the resonance of other parts of the animal's respiratory tract) happen when air flowing past a flap of tissue that is under tension transfers kinetic energy to the flap and thereby induces motion in the flap. The flap then oscillates with a frequency that is a function primarily of its length and tension and of the pressure differential between air upstream of the flap and downstream (Gardner et al. 2001). Importantly for this discussion, the frequency of oscillation in this kind of *hydrodynamic forcing* is independent of the frequency of contraction of muscles except insofar as muscles change the tension in structural features of the syrinx or change the pressure differential in the vocal tract.

**Figure 9.**
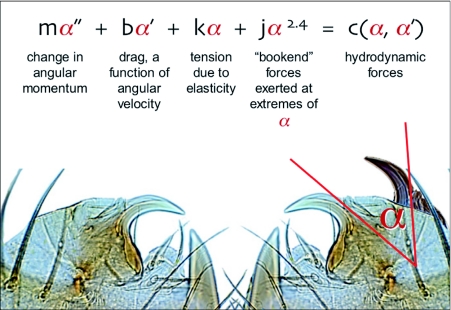
Hydrodynamic forcing model, a hypothetical mechanism that could account for the high-frequency oscillations of the fangs during spitting. See the Discussion for a detailed explanation of the equation, and Video 5 for an animation of the model's output using changes in the mass of the fang, *m*, as the animating variable.

With the data reported in this paper, we cannot with any certainty determine whether the spitting spider's fangs oscillate at high frequency because of asynchronous coupling with muscles or because of hydrodynamic forcing. However, the following is known:

- the muscles responsible for flexing and extending the fang are present in the chelicera (Figure 8) and could either drive the fang oscillations asynchronously or provide and modulate some of the elasticity needed to support oscillation under hydrodynamic forcing;
- asynchronous muscles are, so far, known only from insects (Josephson et al. 2000);
- rapid fluid flow does occur (Table 1), so hydrodynamic forcing is plausible;
- the diameters of the venom duct and sac are much greater than the diameter of the opening of the venom duct on the fang (Figure 8), confirming that the opening must act as a nozzle; and
- the comparatively diminutive size of a spitting spider's fang (Suter and Stratton 2005) makes it particularly well suited to high frequency oscillation because it is the mass that must be accelerated repeatedly during spitting,

Of these, the low probability that asynchronous muscles are found in spiders (2) leads us to believe that hydrodynamic forcing is the more viable model in the current context. Here's how such a model would work. Let α represent the angle of the fang relative to its position at rest, with its long axis approximately perpendicular to the long axis of the chelicera to which it is attached. Because the opening of the venom duct through which the spit is ejected is at a fixed position on the fang (Figure 7), α can also approximate the angle at which spit is ejected relative to its most medial ejection angle (Figure 9). The fang is constrained by its hinge to one axis of rotation, and it is also constrained along that axis of rotation by the mechanics of the fang's other attachments: at the extremes of its excursions (as α approaches either 0° or 70°), the fang's motion is impeded by what we refer to as bookend forces, the resistances of integuments and connective tissues that rise very rapidly with increasingly close proximity to the extremes; and at intermediate positions, the motion of the fang is damped due to friction, the elongation of elastic elements such as tendons, and the forces required to stretch the cheliceral muscles.

For there to be a sustained oscillation, these dissipative forces must be counteracted by forces that accelerate the fang (e.g., the contraction of muscle, the release of previously stored elastic tension, or the change in momentum imparted by the high-velocity ejection of fluid). Symbolically, the model looks like this:

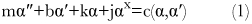

where *m*α″ is the change in angular momentum, the product of the fang's mass and its angular acceleration (note that, as m decreases, the oscillation frequency rises, a likely explanation for the proportionately small size of the fangs in spitting spiders); *b*α′ is drag or friction, dependent upon the fang's angular velocity; *k*α is the elastic tension acting on the fang, at any particular α being the sum of elastic tensions acting either to accelerate or decelerate the fang; and *j*α^x^ represents the bookend forces that constrain the fang's motion at the extremes of its excursions, where *x* > 1 reflects a steep rise in resistance to further change in α. The sum of these forces must equal the hydrodynamic forces, *c*, arising from the forceful expulsion of fluid from the orifice of the venom duct. These hydrodynamic forces will be associated with the rate at which the mass of fluid is ejected (Table 1), but at this point the mass of the ejected material is not known although volume and the velocity are known.

Video 5 shows animated graphical output from Eq. 1, in which *m* (the mass of the oscillating fang) is the animating variable, the other coefficients are held constant, and x is set at 2.0. At every value of *m*, the oscillating nature of Eq. 1 is confirmed, and as *m* varies, the inverse relationship between *m* and oscillation frequency is clear. In the absence of data concerning the values of *m* and the other coefficients, this model remains speculative.

**Video 5.**
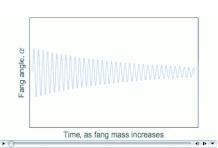
A graphical representation of output from the hydrodynamic forcing model (Figure 9 and Eq. 1). As the animation proceeds, the mass of the fang increases, decreasing oscillation frequency and increasing oscillation amplitude. URL:http://www.insectscience.org/9.62/ref/i1536-2442-9-58-v05.avi

Ultimately, the data and observations reported in this paper do not allow confirmation or falsification of this model in the context of spit ejection. We look forward not only to building and testing scaled up physical models to demonstrate the efficacy of hydrodynamic forcing in spitting spiders but also to measuring the forces represented in Eq. 1 in both intact and anaesthetized spiders.

## Abbreviations

**DICM**: differential interference contrast microscopy
